# A Customizable Tyramide Signal Amplification-Based Multiplex Immunofluorescence Protocol for FFPE Tissues

**DOI:** 10.3390/cimb48050439

**Published:** 2026-04-23

**Authors:** Wenjie Sheng, T. M. Mohiuddin, Chaoyu Zhang, Marwah Al-Rawe, Lutz Konrad, Steffen Wagner, Felix Zeppernick, Ivo Meinhold-Heerlein, Ahmad Fawzi Hussain

**Affiliations:** 1 Department of Gynecology and Obstetrics, Medical Faculty, Justus-Liebig-University Giessen, Klinikstr. 33, 35392 Giessen, Germany; 2 Department of Gynaecology and Obstetrics, Brandenburg Medical School Theodor Fontane, University Clinic Brandenburg, Hochstraße 29, 14770 Brandenburg, Germany; 3 Department of Otorhinolaryngology, Head and Neck Surgery, Justus-Liebig-University Giessen, 35392 Giessen, Germany

**Keywords:** FFPE, mIF, TSA, fluorophore labeling, HRP-conjugated secondary antibody, antibody stripping, heat-induced epitope removal, multi-channel imaging

## Abstract

Formalin-fixed paraffin-embedded (FFPE) tissues represent an invaluable resource for both basic and clinical research due to their stable preservation of tissue architecture and molecular integrity. Multiplex immunofluorescence (mIF) using tyramide signal amplification (TSA) enables the simultaneous detection of multiple antigens within a single FFPE section. Here, we describe a kit-independent and customizable TSA-based mIF protocol that utilizes commercially available horseradish peroxidase (HRP)-conjugated secondary antibodies and tyramide–fluorophore reagents. The method was applied using FFPE endometriosis tissue, targeting estrogen receptor alpha (ERα), progesterone receptor (PR), α-smooth muscle actin (αSMA), CD20 and CD31. Each staining round was followed by heat-induced epitope removal (HIER) of the bound antibodies while preserving covalently deposited signals. Fluorescence imaging was performed using a multi-channel slide scanner with carefully selected fluorophores to enable optical separation between detection channels. Under the conditions described, the protocol enabled clear visualization of maker-specific staining patterns with preserved tissue morphology. This study provides a practical and flexible TSA-based mIF protocol as a qualitative proof of concept, offering an accessible alternative to commercial kit-based approaches. Further studies will be required to establish quantitative performance and a broader applicability across tissue types.

## 1. Introduction

Formalin-fixed paraffin-embedded (FFPE) tissues are abundant clinical specimens, valued for their excellent long-term stability, preservation of cellular morphology and molecular integrity, and linkage to clinical data. These features make FFPE samples an indispensable resource for both prospective and retrospective collections, supporting molecular analysis and targeted therapy in precision medicine and translational research [1,2].

Multiplex immunofluorescence (mIF) has emerged as a cornerstone technique for spatially resolved, high-plex protein detection in FFPE tissues, enabling the simultaneous visualization of multiple antigens in the same tissue. This approach provides a powerful tool for investigating complex cell–cell interactions and immune-profiling within tissue microenvironments, which are essential for assessing disease progression and therapeutic response [3]. In contrast, traditional immunohistochemistry (IHC) offers limited insight into biomarker co-expression and tissue architecture due to its single-marker labeling per tissue section.

**Figure 1:**
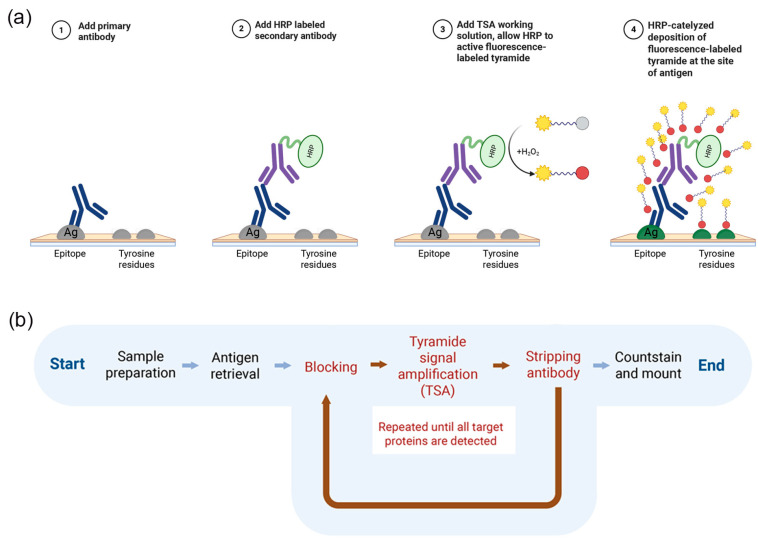
An overview of the TSA-based mIF workflow and signal amplification mechanism. (**a**) A schematic illustration of the HRP–mediated TSA process. Following antigen recognition by a primary antibody and binding of an HRP-conjugated secondary antibody, fluorophore-labeled tyramide is enzymatically activated in the presence of hydrogen peroxide and covalently deposited at or near the antigen sites. (**b**) The TSA-based mIF workflow. Sequential cycles of antigen retrieval, antibody incubation, HRP-mediated tyramide amplification, and antibody stripping are performed to enable the detection of multiple targets within a single FFPE tissue section, followed by nuclear counterstaining. Created in BioRender. Sheng, W. (2026) https://BioRender.com/n62qs16, accessed on 16 April 2026.

Among the current multiplexing approaches, tyramide signal amplification (TSA)-based mIF is the most widely used for FFPE applications. This method relies on horseradish peroxidase (HRP)-mediated catalysis of fluorophore-conjugated tyramide, producing reactive intermediates that covalently bind to tyrosine residues at or near the target antigen. This covalent labeling allows subsequent antibody stripping without signal loss, enabling iterative rounds of staining (Figure 1). Consequently, TSA-based mIF achieves high sensitivity for low-expressing biomarkers while maintaining signal stability, making it suitable for sequential, high-plex biomarker detection in FFPE specimens [4,5].

In recent years, several commercial TSA-based kits have been developed to provide ready-to-use workflows with optimized imaging compatibility across different platforms [5,6]. However, these systems are often costly, offer limited flexibility in fluorophore selection, and rely on proprietary reagents, restricting adaptability for novel experimental designs or resource-limited settings.

Here, we present a kit-independent and customizable TSA-based mIF protocol for FFPE tissues that employs commercially available HRP-conjugated secondary antibodies and tyramide–fluorophore reagents instead of commercial kits. This option provides flexibility for marker panel design without reliance on proprietary systems. As a proof of concept, the protocol was applied to FFPE endometriosis tissues to illustrate its feasibility for multiplex staining and spatial visualization. Fluorescence imaging was performed using a multi-channel slide scanner with carefully selected fluorophores to enable optical separation between the detection channels. The present study focuses on demonstrating the practical implementation and adaptability of its workflow under defined experimental conditions. Further studies will be required to establish its quantitative performance, reproducibility, and broader applicability across different tissue types.

## 2. Materials and Methods

### 2.1. Samples

The FFPE tissue sections and tissue microarray (TMA) sections were obtained from archived samples of patients with endometriosis from the Department of Gynecology and Obstetrics, Justus Liebig University Giessen (Germany). The TMA material was constructed from multiple patient specimens by the clinical pathology department. In total, 50 TMA cores were available and analyzed during this study. Tissue processing and paraffin embedding were performed by the Pathology Department according to standard diagnostic procedures. These samples served as representative specimens to demonstrate the applicability and robustness of the mIF protocol. Importantly, the workflow can be broadly adaptable to other FFPE tissues from different patients and disease contexts. Paraffin blocks were sectioned at a thickness of 4 μm using a microtome. The sections were mounted on adhesive-coated positively charged glass microscope slides and incubated overnight at 37 °C prior to staining. In this study, the term *section* refers to an individual FFPE tissue slice mounted on a glass slide, whereas *slide* represents the glass support carrying the section. Whole-slide imaging refers to the automated scanning of the entire slide area at a defined magnification.

### 2.2. Reagents and Antibodies

1. Primary Antibodies
  • CD20 Monoclonal Antibody (L26) (Invitrogen, Carlsbad, CA, USA).
  • Progesterone Receptor Monoclonal Antibody (R.809.9) (Invitrogen).
  • Estrogen Receptor Alpha (1D5) Monoclonal Antibody (Thermo Fisher Scientific, Waltham, MA, USA).
  • CD31 Monoclonal Antibody (2F7B2) (Invitrogen).
  • Anti-α-Smooth Muscle Actin (ACTA2) Antibody (Sigma-Aldrich, St. Louis, MO, USA).
2. Secondary Antibodies
  • Goat anti-Mouse IgG (H + L) Cross-Adsorbed Secondary Antibody, HRP (Invitrogen).
  • Goat anti-rabbit IgG, HRP-linked Antibody (Cell Signaling Technology, Danvers, MA, USA).
3. Tyramide–Fluorophore Reagents
  The TSA detection system consisted of the following tyramide-conjugated fluorophores:
  • iFluor^®^ 430 Tyramide.
  • Alexa Fluor™ 488 Tyramide.
  • Alexa Fluor™ 546 Tyramide.
  • Alexa Fluor™ 647 Tyramide.
  • iFluor^®^ 750 Styramide.
4. Buffers and Solutions
  The following buffers were used throughout the staining procedure:
  • ROTI^®^Stock 10× TBS reaction buffer (Carl ROTH, Karlsruhe, Germany).
  • Citrate Buffer (10 mM sodium citrate, 0.05% Tween-20, pH 6.0) for antigen retrieval buffer and stripping buffer.
  • PBS and PBST washing buffer (pH 7.4).
  • Normal goat serum (10%) for blocking.
  • Xylene and graded ethanol solutions for deparaffinization and rehydration.
  • Vectashield antifade mounting medium with DAPI.
  All solutions were prepared according to standard laboratory protocols.

### 2.3. Immunohistochemistry Pre-Processing

Prior to multiplex staining, FFPE tissue sections were processed following a standard immunohistochemistry workflow, including deparaffinization, antigen retrieval, and blocking steps. The slides were first incubated at 60 °C for 30 min to melt the paraffin. Deparaffinization was performed using xylene, followed by rehydration through a graded ethanol series (100%, 90%, 80% and 70%). The slides were then rinsed with deionized water and washed in PBST. Antigen retrieval was carried out using heat-induced epitope retrieval (HIER) in a citrate buffer. Slides were heated in a microwave under boiling conditions for 20 min. Slides were then allowed to cool to room temperature. Endogenous peroxidase activity was quenched using 3% hydrogen peroxide, followed by blocking with 10% normal goat serum to minimize non-specific antibody binding.

### 2.4. TSA-Based Multiplex Immunofluorescence

Multiplex immunofluorescence staining was performed using TSA (Figure 1). Five sequential staining cycles were performed, and each cycle targeted a distinct antigen. After each staining round, antibody complexes were removed using microwave-mediated HIER, while the deposited fluorophore signal remained covalently bound to the tissue. Primary and secondary antibodies were diluted in blocking buffer (10% goat serum in PBS). After incubation with the primary antibody and HRP-conjugated secondary antibody, slides were exposed to the appropriate tyramide–fluorophore working solution for signal amplification. Following each staining cycle, antibody stripping was performed using a citrate buffer under microwave heating conditions for 15 min to enable subsequent staining rounds. The optimized antibody–fluorophore combinations and staining order used in this study are summarized in Table 1. To minimize potential steric hindrance and antigen masking caused by excessive tyramide deposition, antibody concentrations and tyramide incubation time were practically optimized to avoid signal oversaturation. In addition, the sequential staining order was carefully designed by considering antigen abundance and spatial localization, such that highly expressed or closely co-localized markers were positioned in earlier staining cycles. These measures were implemented to preserve antigen accessibility and ensure reliable detection in subsequent staining rounds. A detailed step-by-step protocol is provided in the Supplementary Materials (Supplementary Protocol S1), including all experimental parameters and critical steps.

### 2.5. Imaging Acquisition

Fluorescence imaging was performed using a Zeiss Axion Scan.Z1 slide scanner (Zeiss, Jena, Germany) at 20× magnification (Table 2). Fluorescence imaging was performed using separate LED excitation wavelengths and predefined filter sets provided by the Axio Scan.Z1 system; therefore, acquisition parameters are reported as LED wavelengths rather than fluorophore excitation/emission spectrum. Because this platform does not acquire wavelength-resolved emission data, full spectral imaging and computational spectral unmixing were not performed. Instead, fluorescence channel separation was achieved optically through a careful selection of fluorophores with well-separated emission spectra and a channel-specific optimization of the exposure settings. Fluorescence specificity was assessed qualitatively by inspection of individual channels and merged images. Comparing multiplex immunofluorescence localization with corresponding single-plex IHC staining on serial sections was evaluated as well. Under the applied imaging conditions, there was no obvious channel overlap under the applied conditions. Tissue autofluorescence was minimized through antigen retrieval, narrow bandpass filtering, and controlled acquisition parameters.

## 3. Results

### 3.1. Preservation of Tissue Morphology After Sequential TSA Staining

**Figure 2:**
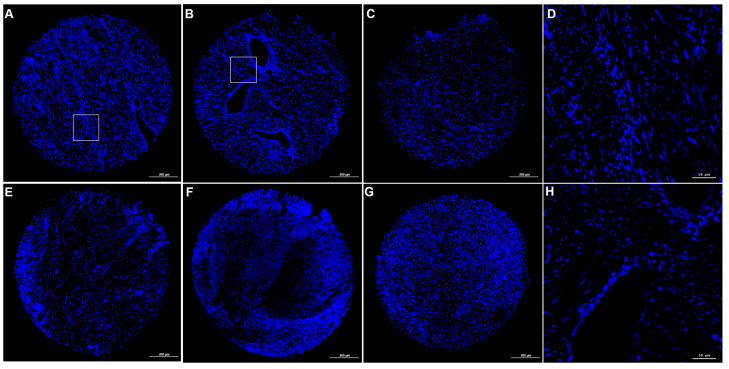
The representative DAPI staining of mIF-processed TMA cores. (**A**–**C**,**E**–**G**) Low-magnification overview images of representative TMA cores showing overall tissue architecture following sequential TSA staining cycles. (**D**,**H**) Higher-magnification views of selected regions from panels (**A**) and (**B**), respectively, illustrating nuclear morphology in different tissue compartments. The regions of interest are indicated by white boxes in panels (**A**,**B**). Scale bar: 200 μm (**A**–**C**, **E**–**G**) and 50 μm (**D**,**H**). These images provide qualitative evidence of a preserved tissue structure under the applied experimental conditions.

Application of the TSA-based mIF workflow to FFPE endometriosis tissues preserved overall tissue morphology and nuclear architecture across the sequential staining cycles. At the overview level, nuclei counterstained with DAPI should appear sharp and evenly distributed, allowing for a visual assessment of the overall tissue integrity following multiplex processing. Representative DAPI overview images from multiple independent TMA cores processed using the TSA-based mIF workflow are shown in Figure 2, indicating that repeated HIER cycles did not result in a visible disruption of the tissue architecture. These observations suggest that the sequential TSA staining procedure maintained the structural integrity of the FFPE sections under the conditions applied in this study.

### 3.2. Qualitative Demonstration of Multiplex Marker Detection

**Figure 3:**
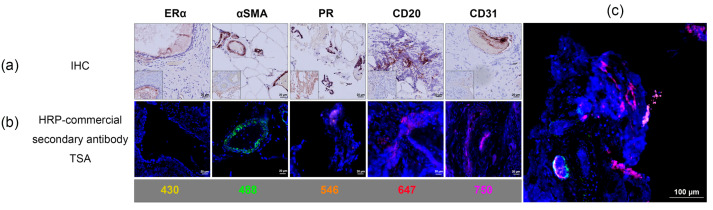
Qualitative demonstration of TSA-based mIF workflow using FFPE endometriosis tissue. (**a**) Representative single-marker IHC staining showing αSMA, PR, CD20, and CD31 expression (brown chromogenic signal). ERα staining was weak or absent. (**b**) Representative TSA-based mIF images from FFPE sections derived from the same tissue block, showing marker-specific fluorescence: ERα (yellow, iFluor^®^ 430), αSMA (green, Alexa Fluor™ 488), PR (orange, Alexa Fluor™ 546), CD20 (red, Alexa Fluor™ 647), and CD31 (magenta, iFluor^®^ 750). The nuclei were counterstained with DAPI (blue). (**c**) A merged multiplex image illustrating a clear visual separation between fluorescence channels and preserved tissue architecture. IHC and TSA-based mIF images are only shown for qualitative illustration and are not a region-matched comparison; therefore, direct spatial comparison is limited. Scale bars: (**a**,**b**) 20 μm and (**c**) 100 μm.

The TSA-based mIF workflow enabled the sequential detection of multiple cellular markers within the same section. Representative chromogenic IHC images and corresponding mIF images are shown in Figure 3. Individual fluorescence channels showed distinct spatial localization within the tissue sections. Nuclear signals were observed in specific cellular compartments, while other markers (ERα, αSMA, PR, CD20, and CD31) displayed cytoplasmic or membrane-associated staining patterns. Vascular structures and scattered immune cells were also identifiable within the multiplex images, demonstrating the ability of the workflow to visualize multiple cellular components within the same tissue section. These observations are presented as qualitative examples and are not intended as quantitative validation.

### 3.3. Multiplex Signal Separation Within Tissue Regions

**Figure 4:**
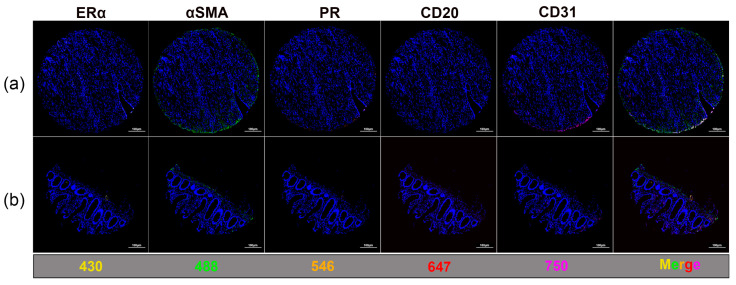
Representative mIF images illustrating multi-channel signal separation within TMA cores. (**a**) Representative images from two TMA cores are shown, selected because they exhibited detectable signals for all investigated markers, allowing for a visualization of the complete multiplex panel within a single tissue context. (**b**) Individual single-channel images—Erα (yellow, 430 channel), αSMA (green, 488 channel), PR (orange, 546 channel), CD20 (red, 647 channel), and CD31 (magenta, 750 channel)—are shown alongside the merged images from two TMA cores processed using the TSA-based workflow and illustrate the spatial localization of multiplex signals within the same tissue regions. Scale bar: 100 μm.

mIF images revealed the visual separation of individual fluorescence channels within the same tissue regions. Representative images from two independent TMA cores are shown in Figure 4, including individual marker channels and merged overlays acquired from identical tissue fields. The two TMA cores shown were selected because they exhibited positive staining for all investigated markers, enabling visualization of the complete multiplex panel within a single field. Distinct fluorescence signals corresponding to ERα (iFluor*^®^* 430), αSMA (Alexa Fluor™ 488), PR (Alexa Fluor™ 546), CD20 (Alexa Fluor™ 647), and CD31 (iFluor*^®^* 750) were observed with spatial distributions consistent with the expected cellular distribution of the corresponding marker. The merged images illustrate the spatial relationships among multiple cellular components within the tissue microenvironment while maintaining a clear separation of individual fluorescence signals. The minimal background fluorescence and absence of visible spectral bleed-through between channels indicated an appropriate fluorophore selection and effective washing conditions during the multiplex staining procedure. These observations are based on qualitative visual assessment.

### 3.4. Imaging Performance and Practical Considerations

Fluorescence imaging was performed using a Zeiss Axion Scan.Z1 slide scanner at 20× magnification. Under these conditions, acquisition of a complete slide required approximately two hours per slide, reflecting the large image size in the multiple fluorescence channels. Although a higher magnification image can provide increased spatial detail, it substantially increases acquisition time and data storage requirements. In addition, prolonged exposure during high-resolution imaging may contribute to fluorophore photobleaching. Therefore, the use of 20*×* magnification represented a practical balance between spatial resolution, imaging throughput, and signal preservation for the routine visualization of multiplex staining.

## 4. Discussion

### 4.1. Performance of the TSA-Based Multiplex Immunofluorescence Workflow

mIF based on TSA has become an important technique for spatially resolved protein detection in FFPE tissues. Unlike conventional IHC, mIF methods allow simultaneous visualization of multiple markers within the same tissue section while preserving morphological context and spatial relationships between cell populations [7]. While TSA is widely reported to enhance detection sensitivity, the aim of the present study was not to directly compare signal intensity with conventional IHC approaches, but rather to establish a flexible, kit-independent multiplex workflow for sequential antigen detection. In the present study, the sequential TSA staining workflow enabled the visualization of fluorescence signals for all investigated markers while maintaining overall tissue morphology. Nuclear counterstaining with DAPI remained clearly visible after multiple staining cycles, suggesting that repeat HIER did not significantly compromise tissue architecture. Previous studies have similarly shown that TSA-based multiplex staining allows for the detection of multiple antigens in FFPE tissues while maintaining structural integrity [8,9]. The staining patterns observed in this study were consistent with the known cellular localization of the investigated markers. ERα and PR are nuclear hormone receptors commonly expressed in the nuclei of glandular and/or stromal cell nuclei [10,11]. αSMA is widely used as a marker of smooth muscle cells and activated stromal fibroblasts, while CD31 is a classical endothelial marker used for identifying vascular structures [12,13]. CD20 is a well-established B-lymphocyte surface marker that identifies B-cell populations in immune infiltrates [14]. These observations are consistent with the expected distribution of the investigated markers and support a qualitative interpretation of staining patterns.

In this study, the workflow primarily enabled a qualitative visualization of the multiple markers within the same tissue section while preserving overall morphology under the applied conditions. However, as comparisons were performed on serial sections and no dedicated specificity controls were included, the interpretation is limited. Therefore, these findings should be considered as a proof of concept rather than a comprehensive validation of staining specificity.

### 4.2. Optimization of TSA Reaction Conditions

Efficient TSA-based multiplex staining requires careful optimization of reaction conditions. The HRP-mediated conversion of labeled tyramide generates highly reactive radicals that covalently bind to proteins near the antigen, resulting in strong signal amplification. While this catalytic amplification greatly increases sensitivity, excessive reagent concentrations may lead to nonspecific signal deposition, whereas insufficient concentrations may reduce detection sensitivity [7]. The optimization of the tyramide–fluorophore dilution was therefore necessary to obtain balanced signal intensities through the sequential staining cycles. In practice, careful optimization of the tyramide concentration and reaction time was critical to balance the signal intensity and background. In preliminary testing, excessive signal amplification occasionally resulted in increased background or potential masking of nearby epitopes, highlighting the importance of stepwise optimization in sequential staining workflows. Buffer composition also plays an important role in maintaining HRP activity and minimizing background fluorescence. In this study, a TBS-based reaction buffer at physiological pH was used to support enzymatic activity, while PBST washing effectively removed unbound reagents. A fresh preparation of the reaction mixture is recommended because the hydrogen peroxide used in the TSA reaction gradually decomposes and may reduce the labeling efficiency [15]. In addition to signal amplification efficiency, the excessive deposition of tyramide may lead to steric hindrance, potentially masking nearby epitopes and affecting subsequent antibody binding. In the present study, this risk was addressed through the careful optimization of reagent concentrations and reaction times, as well as by setting the staining sequence. Although no obvious interference between markers was observed under the applied conditions, such effects cannot be completely excluded, particularly for proteins with high expression levels or close localized targets. Future studies incorporating quantitative co-localization analysis or controlled antigen competition experiments would be valuable to further evaluate the potential signal interference.

### 4.3. Panel Design and Fluorophore Selection

Multiplex imaging experiments require considerable panel design and fluorophore selection to avoid spectral overlap between fluorescence channels. Because some markers may be co-expressed or located in close spatial proximity, fluorophores with well-separated emission spectra characteristics should be selected to facilitate the discrimination between channels. In this study, particular attention was given to markers with potential spatial overlap. ERα and PR are frequently co-expressed within glandular or stromal cell nuclei, while αSMA and CD31 are often localized near vascular structures. To minimize channel interference, fluorophores were selected to maximize the spectral separation between markers with overlapping cellular distributions. Specifically, ERα and PR were labeled with fluorophores exhibiting well-separated emission peaks (iFluor^®^ 430 and Alexa Fluor™ 546), while Asma And CD31 were assigned Alexa Fluor™ 488 and iFluor^®^ 750, respectively. This strategy distributes signals across different wavelength ranges and helps reduce potential channel overlap under the applied imaging conditions. In our experience, fluorophore assignment and staining sequence design were important practical considerations. Markers with a similar localization or high expression required careful separation to avoid visual overlap, particularly in the absence of spectral unmixing. These factors should be taken into account when adapting the protocol to different marker panels. Compared with commercial TSA-based kits, the current workflow offers greater flexibility in antibody and fluorophore selection but requires more manual optimization and user experience. Therefore, while it may be advantageous in research settings, it may be less standardized than kit-based approaches. In addition, considering the excitation and emission spectra of the fluorophores, panel design should also account for the biological localization and potential co-expression of markers within the tissue. Some studies have similarly emphasized that appropriate fluorophore selection and panel design are essential for reliable signal separation and interpretation in mIF [16,17].

### 4.4. Technical Considerations and Limitations

Although this TSA-based mIF protocol offers flexible customization and broad applicability, several technical and practical limitations should be considered. The study was optimized for FFPE tissues, which provide excellent morphological preservation but may exhibit variable antigen retrieval efficiency depending on the fixation duration, tissue type, and processing conditions. These variations can influence epitope exposure and antibody binding efficiency, necessitating the empirical optimization of retrieval conditions for each marker. Endometriosis tissue was selected as a representative model due to its heterogeneous cellular composition, which allows for the evaluation of multiplex staining across different cellular compartments. While this supports the feasibility of the workflow, further validation across diverse tissue types is warranted. Another limitation is the number of markers that can be reliably visualized on a single slide. While the TSA method supports multiple-round staining, repeated antigen retrieval and imaging cycles can lead to gradual antigen degradation or reduced labeling efficiency, particularly for unstable epitopes. Moreover, this protocol is performed manually or semi-manually, which may limit throughput in large-scale studies. However, this approach provides flexibility for the panel design. Future integration with automated platforms may improve scalability. In this study, tissue architecture and antigen localization were evaluated primarily through a comparison of multiplex immunofluorescence with corresponding single-plex IHC on serial sections, providing practical evidence that the applied staining conditions did not result in visible tissue damage. In the present study, the stained slides were routinely imaged within 1–2 days after staining, during which no noticeable signal degradation was observed, indicating good short-term stability. However, long-term signals under extended storage conditions require further investigation. Finally, this study focused primarily on qualitative and spatial visualization. As TSA-based detection involves enzymatic amplification, fluorescence intensity may not be strictly proportional to antigen abundance. Therefore, the current workflow is not intended for precise quantitative analysis, and further validation is required to assess its quantitative performance. Although no unexpected staining patterns were observed, dedicated control experiments would be required to fully exclude potential antibody carryover effects. A further limitation is the lack of dedicated specificity controls for each staining cycle. In particular, controls with no primary or omission controls, as well as systematic validation, were not performed. Therefore, the observed staining patterns are interpreted as qualitative evidence and require further validation in future studies. The integration of multiplex staining with computational image analysis represents a potential direction for future spatial biology studies [18].

## 5. Conclusions

In summary, although the study requires the careful optimization of antigen retrieval and a staining sequence, it presents a flexible TSA-based mIF workflow for FFPE tissues using widely available antibodies and tyramide reagents. The protocol enables sequential detection of multiple markers while preserving tissue morphology and signal specificity. Because the workflow is kit-independent, it provides an accessible alternative to commercial multiplex systems. With the appropriate optimization of antibody panels and fluorophore selection, it can be easily customized for different tissue types and imaging platforms, making it a valuable tool for expanding TSA-based mIF applications in both research and diagnostic contexts.

## Figures and Tables

**Table 1:** Antibodies, staining sequence, and tyramide working dilutions used for TSA-based multiplex immunofluorescence.

Primary Antibody (Target)	Primary Antibody Dilution	Secondary Antibody (HRP-Conjugated)	Position in mIF	Fluorophore	Fluorophore Dilution	Excitation (nm)	Emission (nm)
ERα	1:300	Goat anti-mouse IgG (H + L) HRP	1	iFluor^®^ 430 Tyramide	1:200	433	498
αSMA	1:500	Goat anti-mouse IgG (H + L) HRP	2	Alexa Fluor™ 488 Tyramide	1:100	495	519
PR	1:800	Goat anti-rabbit IgG HRP-linked	3	Alexa Fluor™ 546 Tyramide	1:100	556	573
CD20	1:500	Goat anti-mouse IgG (H + L) HRP	4	Alexa Fluor™ 647 Tyramide	1:100	650	668
CD31	1:400	Goat anti-mouse IgG (H + L)	5	iFluor^®^ 750 Tyramide	1:100	757	779
-	-	-	6	DAPI		360	460

**Table 2:** The multi-channel imaging acquisition parameters.

Marker	Fluorophore	LED Wavelength (nm)	Exposure Time (ms)
ERα	iFluor^®^ 430	430	15
αSMA	Alexa Fluor™ 488	475	5
PR	Alexa Fluor™ 546	555	100
CD20	Alexa Fluor™ 647	630	20
CD31	iFluor^®^ 750	735	300

## Data Availability

The original contributions presented in this study are included in the article/Supplementary Materials. Further inquiries can be directed to the corresponding author.

## Supplementary Materials

The following supporting information can be downloaded at: https://www.mdpi.com/article/10.3390/cimb48050439/s1.

## Abbreviations

The following abbreviations are used in this manuscript:

FFPE	Formalin-fixed paraffin-embedded
mIF	Multiplex immunofluorescence
TSA	Tyramide signal amplification
ERα	Estrogen receptor alpha
PR	Progesterone receptor
αSMA	α-Smooth muscle actin
HIER	Heat-induced epitope retrieval
IHC	Immunohistochemistry
HRP	Horseradish peroxidase
RT	Room temperature
