# Supplementary material for: A Customizable Tyramide Signal Amplification-Based Multiplex Immunofluorescence Protocol for FFPE Tissues
Source: Curr Issues Mol Biol. 2026 Apr 23;48(5):439. doi: 10.3390/cimb48050439 (PMC13204776; doi:10.3390/cimb48050439)
Supplement: Supplementary file 1 [file cimb-48-00439-s001.zip › cimb-4234886-supplementary.pdf]

Detailed TSA-Based Multiplex Immunofluorescence Workflow for FFPE Tissues

S1. Overview

This protocol describes a step-by-step procedure for performing tyramide signal amplification (TSA)-based multiplex immunofluorescence (mIF) on formalin-fixed paraffin-embedded (FFPE) tissue sections. The workflow includes sequential antibody staining, TSA signal amplification, antibody stripping using heat-induced epitope retrieval (HIER), and multi-channel fluorescence imaging.

S2. Materials and Reagents

Primary Antibodies

- CD20 Monoclonal Antibody (L26) (eBioscience™, Invitrogen, Waltham, MA, USA; Cat. no.: 14-0202-82)
- Progesterone Receptor Monoclonal Antibody (R.809.9) (Invitrogen, Waltham, MA, USA; Cat. no.: MA5-14842)
- Estrogen Receptor Alpha (1D5) Monoclonal Antibody, (Thermo Fisher Scientific, Waltham, MA, USA; Cat. no.: MA5-13191)
- CD31 Monoclonal Antibody (2F7B2) (Invitrogen, Waltham, MA, USA; Cat. no.: MA5-15336)
- Anti-α-Smooth Muscle Actin (ACTA2) Antibody (Sigma-Aldrich, St. Louis, MO, USA; Cat. no.: A2547

Secondary Antibodies

- Goat anti-Mouse IgG (H+L) Cross-Adsorbed Secondary Antibody, HRP (Invitrogen, Waltham, MA, USA; Cat. no.: G-21040)
- Goat anti-rabbit IgG, HRP-linked Antibody (Cell Signaling Technology, Danvers, MA, USA; Cat. no.: 7074S)

Tyramide-Fluorophore Reagents

- iFluor® 430 Tyramide (AAT Bioquest, Sunnyvale, CA, USA; Cat. no.: 45096)
- Alexa Fluor™ 488 Tyramide (Invitrogen, Waltham, MA, USA; Cat. no.: B40953)
- Alexa Fluor™ 546 Tyramide (Invitrogen, Waltham, MA, USA; Cat. no.: B40954)

- Alexa Fluor™ 647 Tyramide (Invitrogen, Waltham, MA, USA; Cat. no.: B40958)
- iFluor® 750 Styramide (AAT Bioquest, Sunnyvale, CA, USA; Cat. no.: 45065)

Buffers and Solutions

- ROTI®Stock 10× TBS (reaction buffer; Carl ROTH GmbH + Co. KG, Karlsruhe, Germany)
- Citrate Buffer, 10 mM sodium citrate, 0.05% Tween-20, pH 6.0 (antigen retrieval buffer and stripping buffer; prepared in-house)
- 10× PBS, pH 7.4 (phosphate-buffered saline stock solution; 1.54 M NaCl, 76.8 mM Na_2HPO_4, 23 mM NaH_2PO_4; prepared in-house)
- PBST, pH 7.4 (washing buffer; 154 mM NaCl, 7.68 mM Na_2HPO_4, 2.3 mM NaH_2PO_4, 0.1% [v/v] Tween-20; prepared in-house)
- Normal Goat Serum (blocking buffer, 10%; Abcam, Cambridge, UK; Cat. no.: ab7481)
- Xylene (isomers, deparaffinization reagent; Carl ROTH GmbH + Co. KG, Karlsruhe, Germany)
- Ethanol, ROTIPURAN® ≥99.8%, p.a. (dehydration reagent; Carl ROTH GmbH + Co. KG, Karlsruhe, Germany)
- Vectashield Antifade Mounting Medium with DAPI (Vector Laboratories, Newark, CA, USA; Cat. no.: H-1200)

S3. Equipment

- Fisherbrand™ Multi-Platform Shaker (Thermo Fisher Scientific, Waltham, MA, USA)
- Microwave Oven R242INW (Sharp Corporation, Osaka, Japan)
- Incubator IN75 (Memmert GmbH + Co. KG, Schwabach, Germany)
- Axio Scan.Z1 Slide Scanner (Carl Zeiss Microscopy GmbH, Oberkochen, Germany

S4. Procedure

Note All washing steps were performed on a Fisherbrand™ Multi-Platform Shaker set to 90 rpm at room temperature (RT), unless noted otherwise.

S4.1. Deparaffinization and Rehydration

1. Place slides in a 60 °C oven for 30 min to melt paraffin.
2. Deparaffinize slides sequentially in:

- Xylene (2×, 10 min each)

- 100% Ethanol (2×, 5 min each)
- 90% Ethanol (1×, 5 min)
- 80% Ethanol (1×, 5 min)
- 70% Ethanol (1×, 5 min)

3. Wash slides twice with deionized water for 5 min each, followed by one wash with 1× PBST for 5 min.

Note This protocol was evaluated using FFPE tissues processed under routine diagnostic fixation conditions and is intended for applying to standard clinical specimens.

⚠ **CRITICAL STEP** Ensure slides do not dry out during the entire rehydration process, as drying may cause tissue detachment or high background staining.

S4.2. Antigen Retrieval

4. Place slides in a microwave-resistant jar containing antigen retrieval buffer (pH 6.0).
5. Heat in a microwave oven (100% power) until boiling. Reduce the power to 20% power and keep microwaving for another 20min.

⚠ **CRITICAL STEP** Optimize retrieval conditions according to each primary antibody datasheet.

6. Allow the jar with slides to cool down to RT for at least 20 min.

⏸ **PAUSE STEP** Slides can be kept in citrate buffer at RT for up to 30 min before proceeding to the next step. To accelerate cooling, place the jar in a fume hood with gentle airflow.

S4.3. Blocking Endogenous Peroxidase and Non-Specific Binding

7. Wash slides twice with 1× PBS for 5 min each.
8. Gently dry the area surrounding the tissue section and draw a hydrophobic barrier around each section using a PAP pen. Allow the barrier to dry for approximately 2 min without drying the tissue section.
9. Add 3% hydrogen peroxide (prepared in 1× PBS) to completely cover the specimen and incubate for 15 min at RT in a humidified chamber to quench endogenous peroxidase activity.
10. Wash slides twice with 1× PBS for 5 min each.
11. Add 10% goat serum (prepared in PBS) as a blocking buffer and incubate for 1 h at RT in a humidified chamber.

⚠ **CRITICAL STEP** Ensure the slide is coated evenly with blocking buffer to minimize background. Do not allow slides to dry during blocking.

S4.4. Single-Plex Immunofluorescence and IHC validation

1. Prepare slides following IHC deparaffinization, rehydration, antigen retrieval, and blocking as described in Section 3.1.
2. Incubate slides with primary antibody diluted in blocking buffer for 1 h at RT or overnight at 4 °C in a humidified chamber.
3. Wash slides three times with 1× PBST for 5 min each.
4. Incubate slides with the appropriate HRP-conjugated secondary antibody diluted in blocking buffer for 1 h at RT in the dark.
5. Wash slides three times with PBST for 5 min each.
6. Add appropriate tyramide-fluorophore diluted in reaction buffer and incubate 10 min at RT in the dark
7. Wash three times with 1 X PBST for 5 min each. Optionally rinse once with PBS to remove residual detergent. Counterstain with DAPI, and mount using Vectashield Antifade Mounting Medium with DAPI.

⚠ **CRITICAL STEP** Protect slides from light during all fluorescent steps; avoid drying the tissue surface.

OPTIONAL STEP Include negative controls (primary antibody) to confirm specificity.

8. For validation, perform IHC staining with DAB detection on serial sections using the same primary and secondary antibodies. Compare the localization and distribution between bright-field and fluorescent-field to confirm signal correspondence.

⚠ **CRITICAL STEP** Use identical antigen retrieval and blocking conditions for both IHC and single-plex immunofluorescence staining to ensure valid comparison.

NOTE Systematic IHC validation images are not shown.

S4.5. TSA-based mIF

All primary and secondary antibodies were diluted in IHC blocking buffer (10% goat serum in PBS), all solutions should be prepared freshly and used immediately before application. Sequential staining was performed through five rounds, each targeting a distinct antigen using commercially available primary and HRP-conjugated secondary antibodies and corresponding tyramide-fluorophore reagents. The sequence of staining, antibody dilutions, and fluorophore selection were optimized through trial experiments during protocol development to ensure

balanced signal intensity and to minimize spectral crosstalk. A summary of antibody-fluorophore pairings used in this study is provided in **Table 1**.

S4.6. Primary and Secondary Antibody Staining with TSA reaction

1. Incubate slides with the first primary antibody for 1 h at RT or overnight at 4 °C in a humidified chamber.
2. Wash slides three times with PBST (5 min each).
3. Incubate with the appropriate HRP-conjugated secondary antibody for 1 h at RT.

- Goat anti-Mouse IgG (H+L), HRP (1:100)
- Goat anti-Rabbit IgG, HRP-linked (1:500)

4. Wash slides three times with PBST (5 min each).
5. Prepare the tyramide reaction solution immediately before use by first adding hydrogen peroxide to 1× TBS (pH 7.4) to achieve a final concentration of approximately 0.015% (w/v) in., followed by the addition of the tyramide-fluorophore reagent. Mix gently to avoid foaming. Combine this reaction buffer with the selected tyramide–fluorophore reagent according to the manufacturer’s recommendations.
6. Add the tyramide-fluorophore working solution to cover the tissue and incubate for 10 min at RT in the dark.
7. Wash slides three times with PBST (5 min each).

⚠ **CRITICAL STEP** Keep all fluorescent steps protected from light.

S4.7. Antibody Stripping and Sequential Staining Rounds

8. Place slides in a microwave-resistant jar containing stripping buffer (pH 6.0).
9. Perform HIER using a microwave: heat (100% power) until boiling. Reduce the power to 20% power and keep microwaving for another 15min.

⚠ **CRITICAL STEP** Covalent tyramide solution preserves the fluorescent signal during HIER, enabling repeated cycles

10. Allow the jar with slides to cool down to RT for at least 20 min.

⏸ **PAUSE STEP** After cooling, slides may remain in citrate buffer for up to 30 min; use a fume hood to accelerate cooling.

11. Repeat steps 2-9 for each additional antibody and corresponding fluorophore-tyramide reagents. After the final staining round, proceed directly to step 12 without additional

stripping.

⚠ **CRITICAL STEP** Perform HIER between all staining rounds to remove bound antibodies and prepare the tissue for the next round of staining.

OPTIONAL STEP Include a negative control slide for each staining round.

S4.8. Nuclear Counterstaining and Mounting

12. Wash slides three times with PBS (5 min each), optionally rinse once with PBS to remove residual detergent.
13. Apply Vectashield Antifade Mounting Medium with DAPI and mount coverslips carefully, avoiding bubbles.

⚠ **CRITICAL STEP** Air bubbles interfere with image quality.

⏸ **PAUSE STEP** Mounted slides can be stored at 4 °C in the dark for several weeks.

S4.9. Imaging and Analysis

14. Acquire fluorescence images using a suitable system. Here, we used a multi-channel slide scanner (Zeiss Axio Scan.Z1) at 20× magnification (**Table 2**). Fluorescence imaging was performed using separate LED excitation wavelengths and predefined filter sets provided by the Axio Scan.Z1 system; therefore, acquisition parameters are reported as LED wavelengths rather than fluorophore excitation/emission spectrum. Because this platform does not acquire wavelength-resolved emission data, full spectral imaging and computational spectral unmixing were not performed. Instead, fluorescence channel separation was achieved optically through careful selection of fluorophores with well-separated emission spectra and channel-specific optimization of exposure settings. Fluorescence specificity was assessed qualitatively by inspection of individual channels and merged images. Comparing multiplex immunofluorescence localization with corresponding single-plex IHC staining on serial sections was evaluated as well. Under the applied imaging conditions, no obvious channel overlap under the applied conditions. Tissue autofluorescence was minimized through antigen retrieval, narrow bandpass filtering and controlled acquisition parameter.

⚠ **CRITICAL STEP** Maintain identical imaging parameters across samples for quantitative comparability.

NOTE Repeated scanning or extended exposure to excitation light may reduce signal intensity, particularly for low-abundance markers. Therefore, image acquisition was performed using standardized single-shot scanning conditions.
